# Commentary: Field-based measurement tools to distinguish clonal *Typha* taxa and estimate biomass: a resource for conservation and restoration

**DOI:** 10.3389/fpls.2026.1776560

**Published:** 2026-04-14

**Authors:** Kenneth J. Elgersma, Sydney Aird, Jason P. Martina, Deborah E. Goldberg

**Affiliations:** 1 Department of Biology, University of Northern Iowa, Cedar Falls, IA, United States; 2 Department of Biology, Texas State University, San Marcos, TX, United States; 3 Department of Ecology and Evolutionary Biology, University of Arizona, Tucson, AZ, United States; 4 Department of Ecology and Evolutionary Biology, University of Michigan, Ann Arbor, MI, United States

**Keywords:** clonal plants, experimental design, field identification, morphometrics, spatial dependence, *Typha × glauca*, *Typha latifolia*, *Typha angustifolia*

## Introduction

Hybridization between North American native and non-native cattail species (*Typha latifolia* and *T. angustifolia*) has led to widespread dominance of *T. × glauca*, making the morphological identification of *Typha* critical for conservation and management. Several studies have identified morphological distinctions corresponding to molecular genetic identities, with varied success (Kuehn and White, 1999; Snow et al., 2010; Geddes et al., 2021; Calvo et al., 2025). Ohsowski et al. (2024) used a similar approach but added leaf count - a previously unexplored trait - that they suggest holds strong predictive power of *Typha* genetic ID. While we agree this finding warrants further investigation, we raise questions about the sampling design and analytical methods used in this and similar previous studies (including our own; Calvo et al., 2025). Specifically, we address limitations related to sample size and spatial dependence that influence generalizability, and we offer recommendations for future collaborative research to overcome these limitations.

## Sample size and independence

The small sample size (SSS) problem (Sharma and Paliwal, 2015) is a long-standing problem in Linear Discriminant Analysis (LDA) methods used by Ohsowski et al. (2024) and similar efforts. When the number of potential predictors is large and sample size is limited, “useful” predictors have poor out-of-sample predictive power. In general, for accuracy estimations to be “reasonably close” to reality (Huberty and Olejnik, 2007 p. 309), the *smallest* group’s sample size (n) should be 3–10 times greater than the number of potential predictors (k). Ohsowski et al. start with k=10, so n should be ≥30 for the smallest group–approximately 10-fold greater than their smallest groups (3 for advanced generation hybrids (AGHs); 5 for *T. latifolia*). The risk of SSS problems is amplified by their backward model selection procedure (RFE), which retains only the best predictors (Yates et al., 2023). This optimization process raises the concern that “optimization capitalizes on chance” (Mosteller and Tukey, 1977, p. 769).

To manage SSS problems, Ohsowski et al. use the common approach of cross-fold validation with training and testing datasets. Theoretically, this should safeguard against SSS problems when testing and training data are independent. Unfortunately, that critical assumption was not met due to the clonal nature of *Typha*. The 33 samples Ohsowski et al. used for taxonomic prediction came from 12 transects in 7 wetlands. This spatial structuring resulted in identical genetic IDs within transects for all but 3 samples. All other measured variables were also strongly correlated within transects; we found that “transect” explains most of the variance (typically ~75% and up to 97% of variance) for each. This strong spatial autocorrelation lowers effective sample size below the nominal n=33 and violates the assumption of independence between testing and training data, thereby inflating out-of-sample prediction accuracy (Xu and Goodacre, 2018).

## Assessment with null models

**Figure 1:**
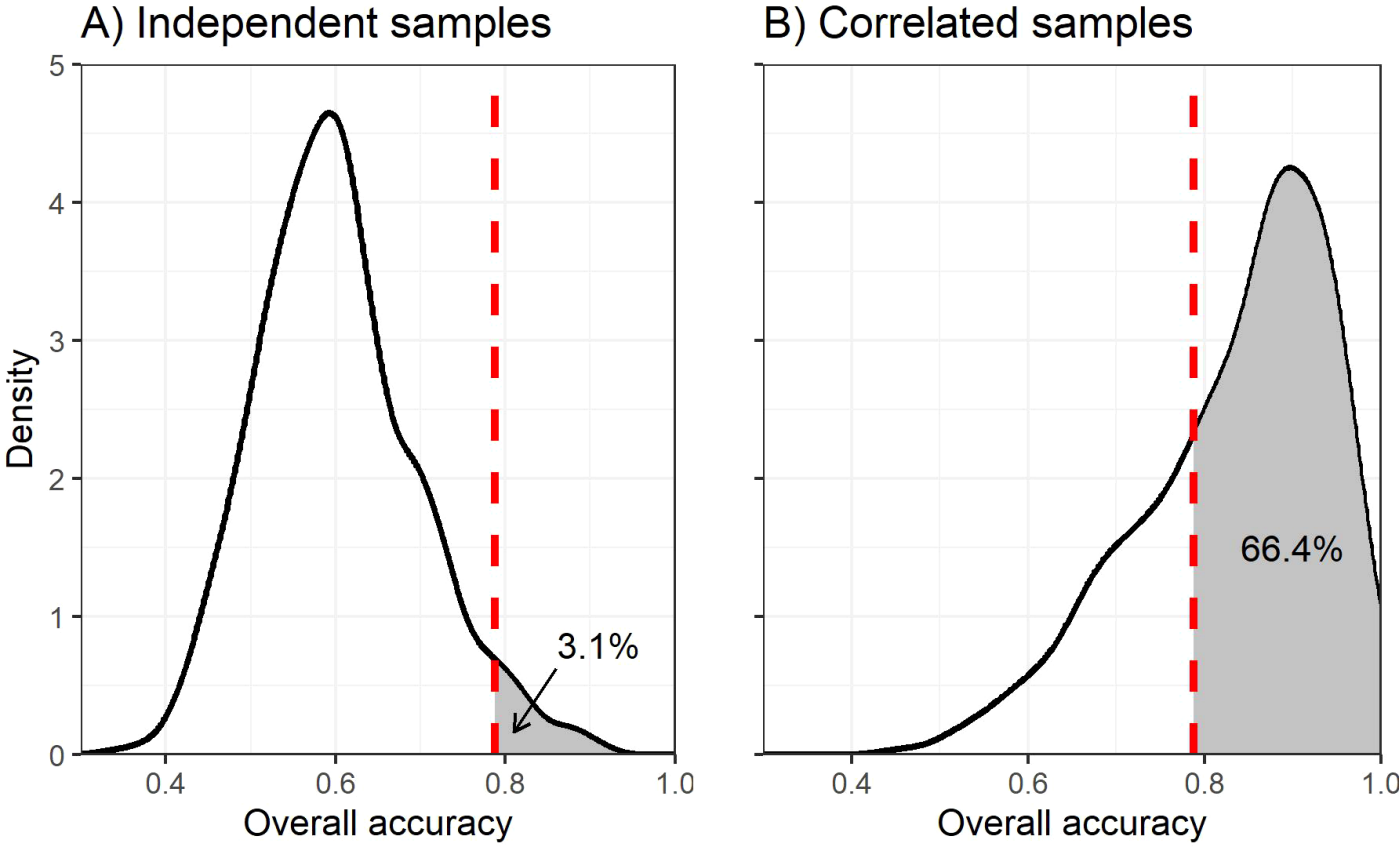
Null distribution for overall prediction accuracy when **(A)** samples are assumed independent or **(B)** spatially correlated within transects. The dashed line represents the overall prediction accuracy observed by Ohsowski et al. (2024), and shaded areas represent the proportion of null model simulations that outperformed Ohsowski et al. (2024).

We constructed two null models to assess the impacts of SSS and nonindependence: one assuming sample independence and one replicating the within-transect correlations observed in the original data. In both cases, we replaced the Ohsowski et al. predictor values with randomly-generated numbers (i.e., “noise”) and used their methods (LDA via RFE with cross-fold validation) to classify the data. We recorded the resulting prediction accuracy and repeated this 1000 times to generate null distributions (**Figure 1**). Assuming sample independence, the observed accuracy of Ohsowski et al. was significantly better than chance (p = 0.031; **Figure 1A**). Although LDA via RFE correctly classified random “noise” 60.1% of the time on average, it rarely outperformed Ohsowski et al. However, when spatial dependence was preserved, the Ohsowski et al. observed accuracy was not significantly greater than chance (p = 0.664; **Figure 1B**). In fact, LDA via RFE correctly classified out-of-sample “noise” 83.3% of the time on average– slightly better than Ohsowski et al. (78.8%). This suggests that the observed spatial autocorrelation allows even random noise to be classified just as accurately as Ohsowski et al.

Our first null model assumes complete independence among samples, such that any observed similarity in morphology is attributable solely to shared taxonomic identity. In contrast, our second null model assumes that samples are spatially-correlated and therefore any similarity in morphology is due to being in the same transect. Reality likely lies somewhere between these two scenarios, but unfortunately it is difficult to determine precisely where because transect ID and taxonomic ID are nearly perfectly confounded. As a result, it is also not currently possible to know whether the Ohsowski et al. LDA model predicts taxonomic ID better than random chance or not.

## Discussion

Our null models show the importance of sample independence for LDA and other multivariate techniques. Sampling clonal plants creates at least two sources of spatial non-independence: genetic and environmental. Travis et al. (2011) showed that ramets are frequently genetically identical up to 20 m apart (and occasionally up to 90 m), suggesting that samples should be collected at least 20m and ideally ~100 m apart. Additionally, non-independence can arise for traits that show environmental phenotypic plasticity, e.g. wider, longer, and more numerous leaves in nutrient-enriched environments (Knops and Reinhart, 2000). Together, these observations suggest it is better to sample few individuals from many wetlands, rather than many individuals in few wetlands. In fact, the ideal sampling design may involve collecting one individual per taxonomic group per wetland environment, across as wide a range of environmental conditions as possible.

This idealized sampling scheme is costly, labor-intensive, and would likely reduce sample sizes. However, the relatively positive outcomes from our first null model demonstrate that small sample size is less of a concern than non-independence. We recommend that future research prioritizes sample independence over sample size. Such a design is also ideally suited for a broad collaborative effort. Individual researchers using identical sampling protocols could collect a few samples from just a few locations, then combine samples to assess the ability of morphological traits to distinguish *Typha* taxa. A broad collaboration can overcome SSS and sample independence problems to seek generalizable, useful morphological traits.
